# The Hazard Perception for the Surrounding Shape of Warning Signs: Evidence From an Event-Related Potentials Study

**DOI:** 10.3389/fnins.2018.00824

**Published:** 2018-11-08

**Authors:** Qingguo Ma, Xiaoxu Bai, Guanxiong Pei, Zhijiang Xu

**Affiliations:** ^1^Institute of Neural Management Sciences, Zhejiang University of Technology, Hangzhou, China; ^2^School of Management, Zhejiang University, Hangzhou, China; ^3^College of Information Engineering, Zhejiang University of Technology, Hangzhou, China

**Keywords:** warning signs, surrounding shapes, Event-Related Potentials (ERP), neural industrial engineering (NeuroIE), neuromanagement

## Abstract

Surrounding shape is a very important component of warning signs. Unlike colors, signal words, and pictorials that can directly convey the surface meaning, the surrounding shapes of warning signs convey warning information somewhat obscurely. Most of the researchers who studied this topic investigated the individuals' hazard perception of the surrounding shapes of warning signs by using questionnaires. In addition, the scholars' points about the role of the surrounding shapes are inconsistent. This study, therefore, decided to use Event-Related Potentials (ERP) technology to explore the impact of the shapes on the perception of warning signs to find the evidences of the hazard perception of the shapes from the electrophysiological perspective. Using the Oddball paradigm, we found four components caused by different shapes of warning signs. Specifically, P200 amplitude characterizes the attraction to attention of surrounding shapes in the early automatic perception stage, the N300 components represented the emotional valance and arousal level, the P300 and the LPP connoted uneasy/unsafe information and reflected the inhibition strength on the uneasy/unsafe information. Experimental data indicated that the shape of UPRIGHT TRIANGLE had larger arousal strength and more negative valence than the shape of CIRCLE. People get stronger negative information from the UPRIGHT TRIANGLE shapes than from the CIRCLE. This finding might be helpful for designing the surrounding shapes of warning signs.

## Introduction

Visually, it is an effective way to communicate hazard information through warning signs. Hazard information delivered by warning signs are considered necessary in a variety of industries. The basic elements of the warning signs are composed of signal words, colors, surrounding shapes, and pictorial symbols (Young, 1998). It has been found that the surrounding shape was an important factor to affect the perception of hazard information and the reaction time to avoid hazard, specifically, the INVERTED TRIANGLE got higher warning score and made people react faster than did the shape of CIRCLE. The Chinese word “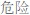 (DANGER)” surrounded by INVERTED TRIANGLE shape constituted the most effective warning sign to deliver the hazard information (Wang et al., 2008). The relations between individual's variables (such as sex, age, education, familiarity with warning sign, and risky preference) and the perception of the warning signs were been studied (Rogers et al., 2000). It was revealed that the colorful signs might be more useful than monotone signs or signs with fewer colors for young children (Siu et al., 2015). In addition to the studies on the elements of the warning signs, some researches paid attention to the theory of warning signs. For instance, the communication-human information processing (C-HIP) model of warning signs suggested that there were four stages of information processing before action when people were seeing warning signs. The four stages included attention, comprehension, attitudes and beliefs, and motivation (Wogalter et al., 2005; Shang et al., 2015). By using Event-Related-Potential (ERP) techniques to study this issue, the researchers found that there were only two stages occurred in brain about dealing with the signal words in safety signs. The first stage was an early automatic cognitive process of consciousness for the risk in signal words, represented by P200; the second stage was the late controlled process in which the risk level that the signal words included was evaluated, represented by LPP (Ma et al., 2010).

As a controversial part of a warning sign, the surrounding shapes, unlike signal words and pictorial symbols that can directly convey the meaning of their surface, cannot deliver warning information directly. The warning effect that shapes convey is somewhat vague. So, most of the investigations about surrounding shapes are usually used together with other elements, such as signal words and pictorial symbols. In earlier studies, Michael A. Rodriguez revealed that written labels surrounded by a shape resulted in higher compliance than the labels without surrounding shape, and the color had significant effects only when used combination with shapes. So the surrounding shape is an important component of the warning sign (Rodriguez, 1991). But until now, there is no consistent answer to the question of whether the surrounding shapes can contribute to warning effect. Rui-feng Yu et al. suggested that some shapes may have a strengthening effect for hazard communication, such as INVERTED TRIANGLE and UPRIGHT TRIANGLE, while some others have a weakening effect, such as RECTANGLE (Yu et al., 2004). Michael W. Riley et al. considered that the INVERTED TRIANGLE was the perfect warning indicator among the shapes, whereas the CIRCLE was generally not perceived as a warning shape (Riley et al., 1982). S. David Leonard deemed that the shape or other graphical configurations might help better than color to convey different levels of risk (Leonard, 1999). Contrary to the above points, Brewster demonstrated that surrounding shapes can make the signal word more difficult to read when approached from a certain perspective (Brewster, 1995). Stephen L. Young thought that surrounding shapes made warning signs difficult to read (Young, 1998). Up to now, some studies evaluated the surrounding shapes alone, and some others investigated the surrounding shapes with a variety of elements together, such as warring words, pictorial symbols, and colors. The shapes that have been studied covered UPRIGHT TRIANGLE, INVERTED TRIANGLE, DIAMOND, CIRCLE, and RECTANGLE, etc.

There are some researches aiming at context-free geometric shapes or the outline of a semantically neutral object. In detail, Christine L. Larson et al. had used an Implicit Association Test to examine associations between three shapes (downward- and upward-pointing triangles, circles) and pleasant, unpleasant, and neutral scenes. They found that even very simple context-free geometric shapes have been shown to signal emotion, conveying affect associated with perception of threat or unpleasantness. Where the downward-pointing triangles are quickly classified as unpleasant, the circle is considered pleasant. And they extend the support for the configural hypothesis of affect perception to the domain of implicit cognition (Larson et al., 2012). Moshe Bar et al. compared amygdala response to objects whose semantic meaning is emotionally neutral but with different types of contour by using fMRI, the result was that the amygdala shows significantly more activation for the sharp angled objects compared with their curved counterparts, and experiments had verified that this activation is related to contour type (Bar and Neta, 2007). Indeed, the amygdala has been shown to respond to implicit, non-conscious cues of threat, so that these sharp visual elements can increase the sense of threat and danger (Whalen et al., 1998).

But almost all the previous studies took interview and/or questionnaire to record the subjects' perception of arousal strengthen of the elements of warning signs, and considered these results as the effectiveness of the elements of warning signs (Wang et al., 2008). These approaches are largely influenced by the participants' intuition and daily experience, so that, in fact, it was a result of immunization. In the past, there were only a few studies which used physiological techniques, such as the electroencephalogram (EEG) and ERP, to study the effectiveness (Ma et al., 2010). As we mentioned above, the shapes had not the strong readable meaning as same as the warning words and pictorial symbols had. So far, the effectiveness of the shapes in warning signs were still controversial. This study, therefore, tried to apply ERP to examine the effectiveness of the shapes and the process of perception and cognition of the shapes.

ERP is a kind of electrophysiological techniques which directly measure the subject's perception and cognition process of the stimulus. In most of the ERP experiments about warning signs, participants were asked to estimate the perceived hazard level of a warning sign whenever they saw the sign, and at the same time, their EEG were recorded (Ma et al., 2010). Only a few studies let subjects finish the task of estimation on hazard level and hiding the real purpose of the experiment. This study used an Oddball paradigm in which the subjects had to press a key when the non-warning-sign picture, such as umbrella, was presented, whereas they had no task when the picture of a warning sign was presented, so as to avoid a “relevance-for-task” effect (Carretié et al., 1997b; Yuan et al., 2007).

### P200

P200 is an early positive ERP component with a peak latency from 100 to 200 ms, and is considered to be an indicator at the boundary of unconsciousness and consciousness, and it seems to be an attention bias occurring automatically (Huang and Luo, 2006), and to be sensitive to the emotionality of stimuli (Wang et al., 2012). A Previous study had shown that P200 was associated with early detection of threatening stimuli, such as frightful images (Correll et al., 2006). And the enhanced P200 amplitude showed the distribution of attention resources to significant stimuli (Carretié et al., 2001). In implicit emotional task, a frontal P200 was elicited in all conditions, meaning rapid detection of typical stimulus features. A previous study found that the smaller P2 amplitude was observed for the extremely negative (EN) condition than for the moderately negative (MN) and neutral conditions. What is more, the study observed shorter P2 latency for the EN condition than for the MN and neutral conditions, suggesting that people perceived EN things more faster than others (Yuan et al., 2007).

### N300

It was suggested that the N300 component of the ERP constituted a useful tool for studying the emotional reactions to visual stimuli, and it was less influenced by cognitive variables than P300. N300 showed larger amplitudes related with the activating positive visual stimuli, especially at parietal sites (Carretié et al., 1997b). So the N300 has been proved in previous studies that it represented an ability to constitute a more suitable component for distinguishing the different affective characteristics of visual stimuli, and its highest amplitude was in response to activating ones (Carretié et al., 1997a).

### P300

P300 is a positive ERPs component with a peak latency between 300 and 400 ms after stimuli, and it widely scattered in the brain area with the amplitudes increase from front to back. It is associated with evaluative categorizations, and reflects the cognitive evaluation of stimuli's meaning (Ito et al., 1998; Huang and Luo, 2006), and differentiates emotional or threatening stimuli from neutral stimuli during active evaluation (Correll et al., 2006; Muñoz and Martín-Loeches, 2015). It was found that the P300 elicited by EN stimuli had shorter latencies when comparing with the stimuli in MN and neutral conditions, showing that the meaning of EN stimuli were preferentially analyzed and evaluated, and the P300 amplitude evoked by the EN stimuli was the smallest among the three conditions, and the largest by the neutral stimuli (Yuan et al., 2007). Studies also indicated that posterior P300 is an index of an inhibition of task-irrelevant information, and also represents later conscious categorization, decision-making and premotor response-related activities (Donchin, 1981; Goldstein et al., 2002). The amplitude of P300 may, therefore, reflected the degree that people resisted the irrelevant task (Yuan et al., 2007).

### LPP

Late positive potential (LPP) is an ERP component maximal over central-parietal regions occurring between 300 and 700 ms after stimuli onset, and is modulated by the emotional intensity of a stimulus (Brown et al., 2012). It was proved that the hazard level that the warning words contained was represented by LPP compoment (Ma et al., 2010).

Based on foregoing studies on warning signs and ERP components, we speculate that the individual's perception and cognitive process of different surrounding shapes of the warning signs are different. And the differences can be reflected by the ERPs components (P200, N300, P300, and LPP). We suppose that the different shapes of warning signs might evoke the components of P200, N300, P300, and LPP. In particular, the P200 amplitude characterizes the attraction to attention of surrounding shapes in the early automatic perception stage, the N300 component represents the emotional valance and arousal level, the P300 connotes uneasy/unsafe information and reflects the degree that people resisted the irrelevant task, and the LPP reflects the emotional intensity of a stimulus. According to the inquiry from small-scale questionnaire, we guess that it is more effective for UPRIGHT TRIANGLE to be the surrounding shape of the warning signs to convey hazard information than for CIRCLE.

## Methods

### Participants

A total of 20 Chinese students from Zhejiang University enrolled in this experiment, data from one participant were excluded because of excessive recording artifacts. The remaining 19 participants ranged from 20 to 27 years old (mean = 23.79, *SD* = 2.04). All reported normal or corrected-to-normal vision and had no history of current or past neurological or psychiatric illness. Informed consent was obtained from all participants, and this study was approved by the Ethics Board of Zhejiang University Neuromanagement Laboratory. At the end of each experiment, the participant got a fee of ¥ 40. And finally, valid data of 19 subjects were obtained for analysis.

### Experimental materials

**Figure 1:**
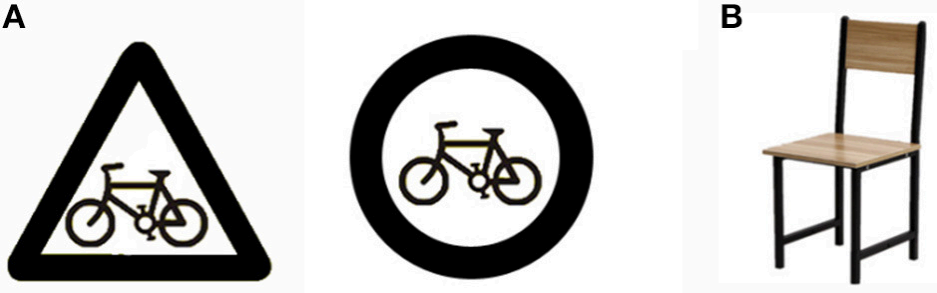
Pictures are stimuli showed to participants. For examples, **(A)** shows the non-target stimuli for neutral indicative warning signs with two conditions (UPRIGHT TRIANGLE vs. CIRCLE) and **(B)** shows the target stimulus for chair.

In this study, an Oddball paradigm was used, and the target stimuli were 7 kinds of tables and chairs. Each of target stimuli was presented 3 times, so there were total 21 trials for target stimuli. Non-target stimuli included two kinds of shapes (CIRCLE vs. UPRIGHT TRIANGLE) with 14 kinds of pictorial symbols inside the surrounding shapes. Each non-target stimulus appeared also 3 times, so each condition (CIRCLE or UPRIGHT TRIANGLE condition) contained 42 trials, and there were total 84 trials for non-target stimuli. The shapes were made of white background with a thin, inked black line border. The pictorial symbols inside the signs were selected from neutral indicative warning signs, such as the direction of exit, to exclude other influence, and the color of the surrounding shapes was black too. All the stimuli materials were processed by Photoshop software to keep their quality, aspect, gray scale, and pixel the same (see Figure 1 as an example, see the Supplementary Figure 1 for details). All stimuli were presented at random.

### Experimental procedure

**Figure 2:**
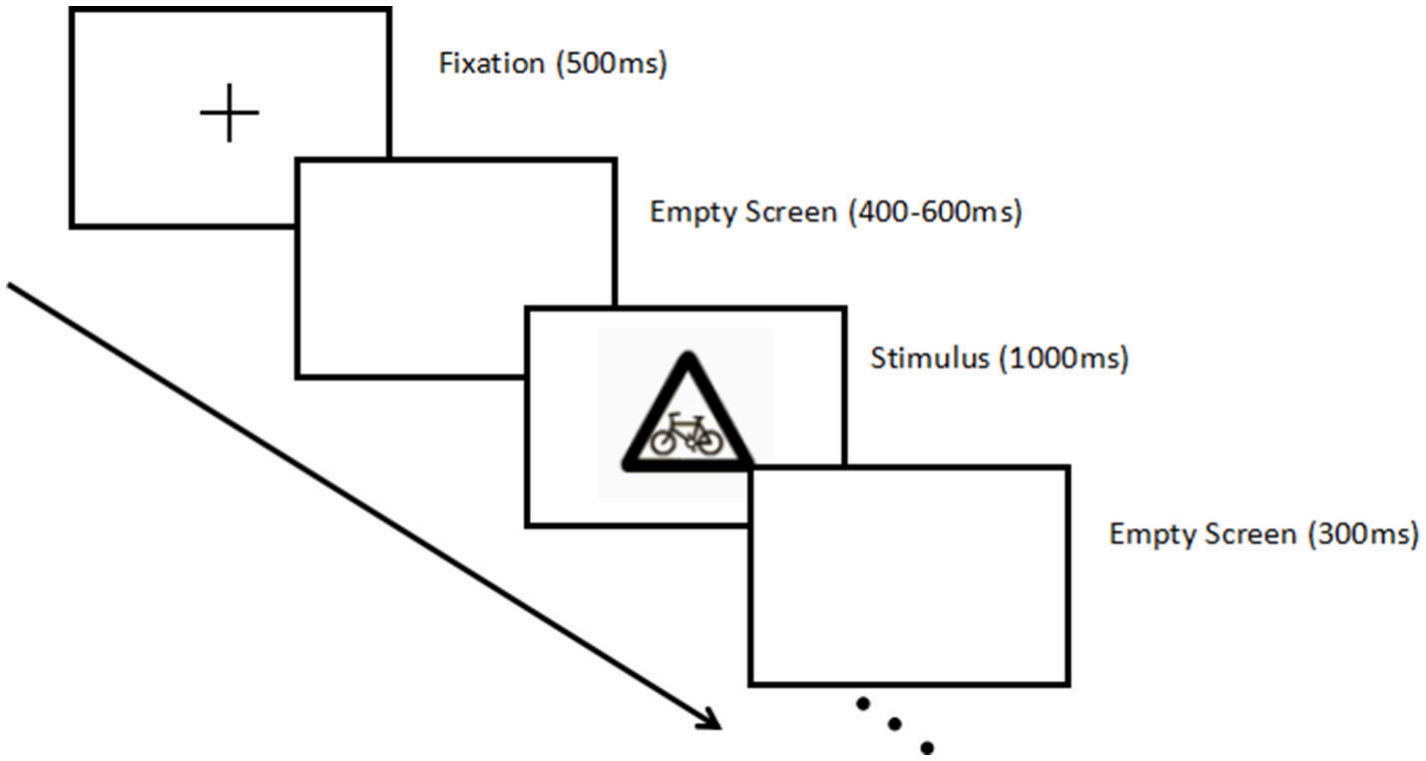
Illustration of the stimulus paradigm applied.

The participants sat comfortably in a dimly lit, sound-attenuated and electrically shielded room, and the keypad was fixed on the chair. The stimuli were presented centrally on a computer screen at a distance of 100 cm away from the subjects. Firstly, the experimenter introduced the instruction and conducted a brief practice exercise. The experiment was about 5 min, meanwhile, the participants' EEG were recorded throughout the experiment. The experiment was consisted of 105 trials. At the beginning of each trial, a fixation appeared as a cue for 500 ms, after a blank screen presented for a duration varying randomly between 400 and 600 ms, followed by the stimuli materials which was presented at the center of screen for 1,000 ms. Participants were asked to respond as quickly as possible with the thumb on the “1” button on the keyboard when the target stimuli occurred. The responding hand was counterbalanced across participants. At the end of a trial, an empty screen was presented about 300 ms (see Figure 2).

When the participants complete the experiment, they were asked a question: Which surrounding shapes, CIRCLE or UPRIGHT TRIANGLE, of the warning signs do you think is more vigilantly?

### ERP data acquisition

EEG was continuously recorded (band-pass 0.05–100 Hz, sampling rate 500 Hz) with the Neuroscan Synamp2 Amplifier (Scan 4.3.1; Neurosoft Labs, Inc., Sterling, Virginia, USA). And using an electrode cap with 64 Ag/AgCl electrodes, according to the international standard 10–20 system. The left mastoid served as on-line reference. EEGs were off-line re-referenced to the average of the left and the right mastoids. The electrode on the cephalic location was applied as ground. Vertical and horizontal electrooculogram (EOG) were recorded with two pairs of electrodes, a pair of electrodes placed between the two sides of the eye (horizontal EOG) and the other placed upper and lower left eye 10 mm. All EEG electrode impedances were maintained below 5 kΩ.

### ERP data analysis

In the off-line analysis, the EEGs under different surrounding shapes were averaged. Ocular artifacts were corrected with an eye-movement correction algorithm provided by Neuroscan 4.3 software. The ERPs were digitally filtered using a low pass filter at 30 Hz (24 dB/octave) and corrected to the baseline. EEG recordings were extracted from −200 to 800 ms and time-locked to the onset of stimulus, and the whole epoch was baseline-corrected by the 200 ms interval prior to stimulus onset and peak-to-peak deflection exceeding ± 80 μV were excluded. More than 30 sweeps for each condition remained.

The mean amplitude in the time window of 170–220 ms after the stimuli onset was calculated for P200, the time window of 285–325 ms for N300, the time window of 220–300 ms for P300, and the time window of 450–700 ms for LPP. In order to analyze the effects of these components, we selected the five electrode points (F1, F3, FZ, F2, and F4) in the frontal area to analyze P200 and N300. P300 was analyzed by selecting six electrode points (C3, CZ, C4, CP3, CPZ, and CP4), and nine electrodes (C3, CZ, C4, CP3, CPZ, CP4, P3, PZ, and P4) in the central and parietal area were selected for LPP.

In this experiment, repeated measurements were used to measure variance analysis with two factors: Surrounding Shapes (CIRCLE vs. UPRIGHT TRIANGLE) and the Electrodes for each of the four components.

## Results

### Behavioral results

According to statistics, only one participant considered the shape of CIRCLE was more alert than the UPRIGHT TRIANGLE. Others had the opposite view.

### Event-related potentials results

#### P200

**Figure 3:**
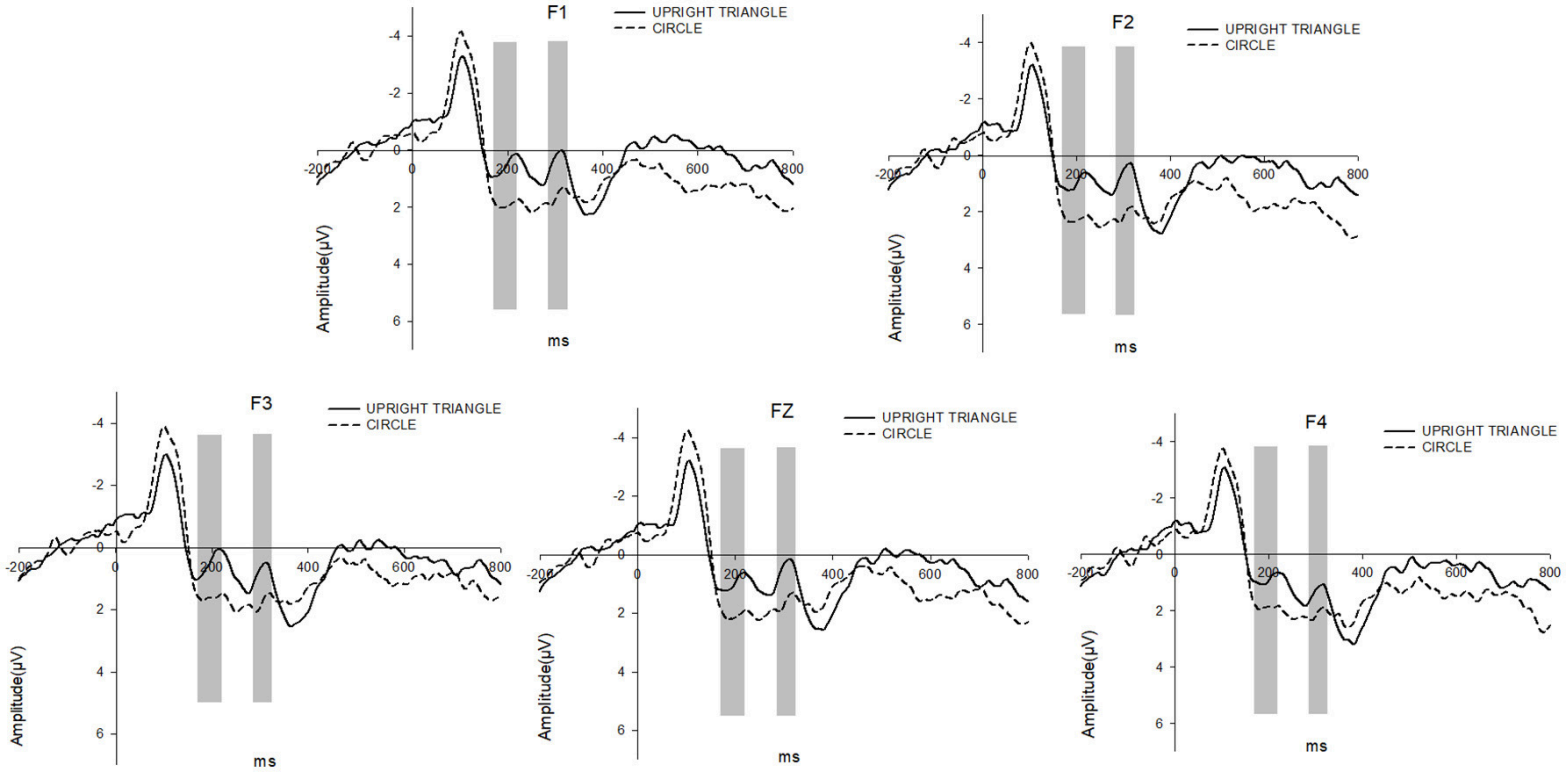
c P200 and N300 were elicited by UPRIGHT TRIANGLE condition (solid line) and CIRCLE condition (dashed line), respectively. The time window for P200 is 170–220 ms and that for N300 is 285–325 ms.

The 2 (Surrounding Shape: CIRCLE vs. UPRIGHT TRIANGLE) × 5 (Electrode: F1, F3, FZ, F2, and F4) repeated measure ANOVA on P200 revealed a significant main effect for the Surrounding Shape [*F*_(1, 18)_ = 6.616, *p* < 0.05]. The P200 amplitude elicited by UPRIGHT TRIANGLE (mean = 0.878 μV) was significantly smaller than that by CIRCLE (mean = 2.029 μV). But there was no significant difference between the electrodes. And the interaction between the Surrounding Shape and the Electrode did not show a significant difference (see Figure 3).

#### N300

As to N300, the 2 (Surrounding Shape: CIRCLE vs. UPRIGHT TRIANGLE) × 5 (Electrodes) repeated measure ANOVA revealed a significant main effect for the Surrounding Shape [*F*_(1, 18)_ = 9.630, *p* < 0.05]. And there was a greater N300 amplitude in the condition of UPRIGHT TRIANGLE (mean = 0.643 μV) when compared to the condition of CIRCLE (mean = 1.855 μV). But no significant differences between the electrodes were found. The interaction between the Surrounding Shape and the Electrode did not show a significant difference (see Figure 3).

#### P300

**Figure 4:**
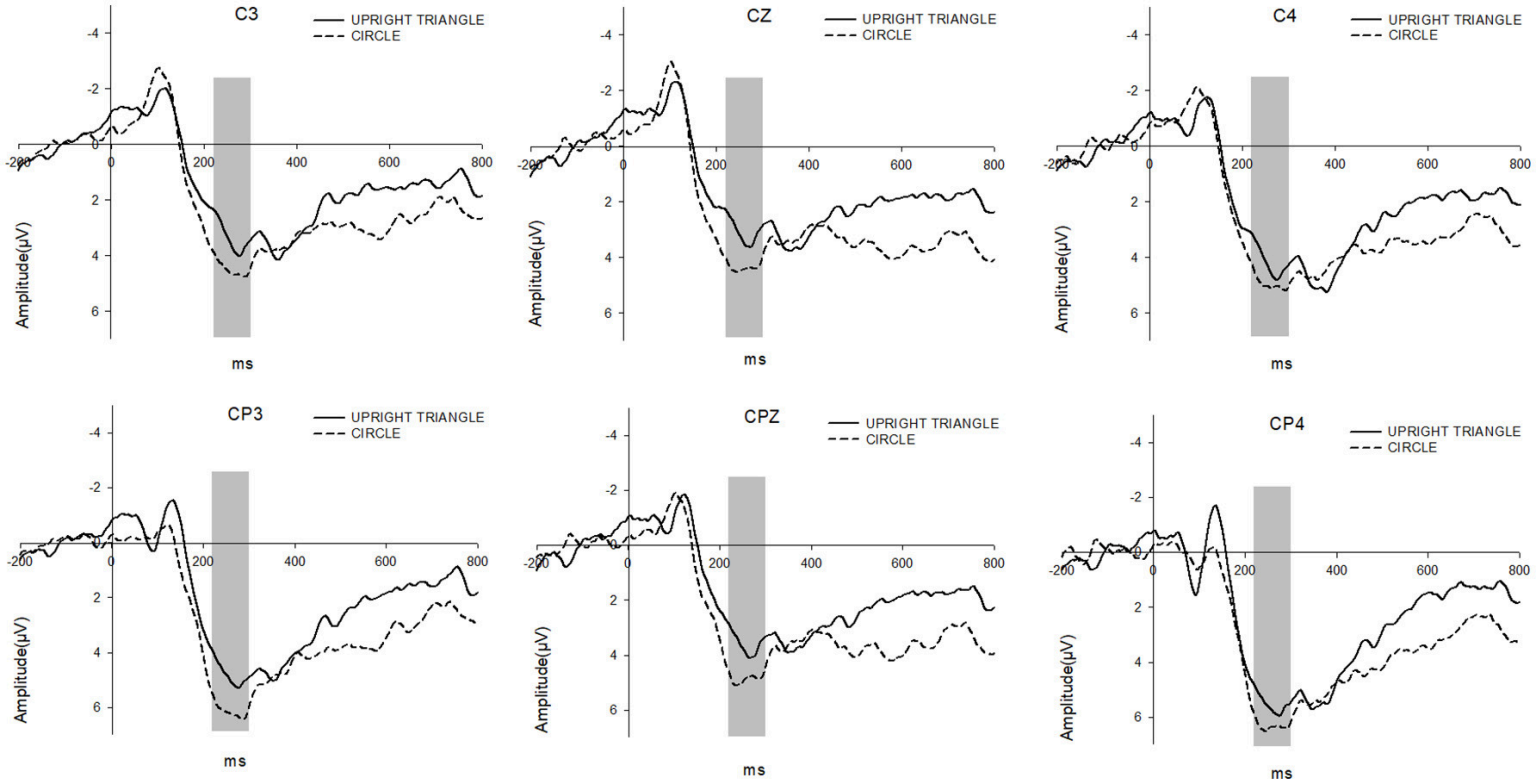
P300 were elicited by UPRIGHT TRIANGLE condition (solid line) and CIRCLE condition (dashed line), respectively. The time window for P300 is 220–300 ms.

The 2 (Surrounding Shape: CIRCLE vs. UPRIGHT TRIANGLE) × 6 (Electrode) repeated measure ANOVA on P300 revealed a significant main effect for the Surrounding Shape [*F*_(1, 18)_ = 14.588, *p* < 0.005]. A remarkably larger P300 was found for the shape of CIRCLE (mean = 5.363 μV) when compared to the shape of UPRIGHT TRIANGLE (mean = 4.185 μV). And there was also a significant difference between the electrodes [*F*_(5, 90)_ = 6.353, *p* < 0.005]. But the interaction between the Surrounding Shape and the Electrode did not show a significant difference (see Figure 4).

#### LPP

**Figure 5:**
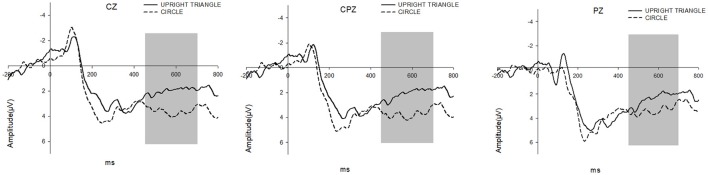
LPP were elicited by UPRIGHT TRIANGLE condition (solid line) and CIRCLE condition (dashed line), respectively. The time window for LPP is 450–700 ms.

The 2 (Surrounding Shape: CIRCLE vs. UPRIRHT TRIANGLE) × 9 (Electrode) repeated measure ANOVA on LPP showed a significant main effect for the Surrounding Shape [*F*_(1, 18)_ = 9.947, *p* = 0.005]. Comparing to the shape of CIRCLE (mean = 3.398 μV), UPRIGHT TRIANGLE (mean = 2.110 μV) showed an evident smaller LPP amplitude. But there no other significant effects could be demonstrated about the Electrode and the interaction between the Surrounding Shape and the Electrode (see Figure 5).

## Discussion

In previous studies on warning sign, the viewpoints about the effectiveness of the surrounding shapes were most divisive (Riley et al., 1982; Leonard, 1999; Yu et al., 2004). Stephen L. Young argued that surrounding shapes made warning signs difficult to read, and its profits might be less than its defects (Young, 1998), while Yu et al. (2004) as well as Riley et al. (1982), by using the behavior experiments or questionnaire, gave a clear order of the warning levels that were delivered by different surrounding shapes. The results of the current study, from the perspective of the cognitive neuroscience, provided evidence to prove that ERP components related to attention (represented by P200), valence and arousal (represented by N300) are affected by the UPRIGHT TRIANGLE border, and more uneasy/unsafe information which was inhibited more strongly at the cognitive level (represented by P300 and LPP) when compared with CIRCLE.

By using ERP with high temporal resolution, we found four ERP components might be considered as the physiological indicators of the perceived and cognitive processes after seeing the warning signs. In details, firstly, according to the previous study (Yuan et al., 2007) which showed the frontal P200 activation was an automatic and rapid detection to the threatening content, and more attention resource was recruited in this process when compared with neutral condition, our result of P200 suggested that the UPRIGHT TRIANGLE border was detected automatically and more quickly when compared to the CIRCLE border. Besides, the current study provided direct evidence to support above suggestion: the P200 latency for UPRIGHT TRIANGLE border was significantly shorter than for CIRCLE border, meaning that the response to UPRIGHT TRIAANGL border was faster than to CIRCLE. This electrophysiological founding supported the previous behavioral research which found that people reaction time to TRIANGLE was less than to CIRCLE (Wang et al., 2008).

Secondly, the N300 was an emotion-sensitive component. Unlike P300 which was mainly evoked by the cognitive processing, the N300 was less influenced by cognitive variables (Carretié et al., 1997b). L. Carretié and colleagues in their studies (Carretié et al., 1997a,b) used a distracting task (to focus on the “correspondence” or different themes of stimuli) to disguise the real aim of the experiments to avoid the “relevance-for-task effect,” and found that the “activating and repulsive” stimuli (such as human remains) elicited more negative N300 in frontal and parietal regions when compared to the “activating and attractive” stimuli. The current study which also adopted the distracting task (to focus on the target stimuli, such as tables and chairs) found that the UPRIGHT TRIANGLE border elicited larger N300 amplitude in frontal region than did the CIRCLE border, whose logic was very similar to studies (Carretié et al., 1997a,b), suggesting that the UPRIGHT TRIANGLE border delivered more uncomfortable/uneasy/unsafe felling to subjects when compared with the CIRCLE border, i.e., the UPRIGHT TRIANGLE has higher negative valence than the border of CIRLCLE has.

Wei et al. (2011) compared the differences in neural response to negative or positive words between the group in which individuals had traumatic experience in earthquake and the group having no the experience. They found that more negative N300 effect for negative words than for positive ones in the earthquake group, whereas no this effect was found in the control group (having no experience in earthquake). The result showed that more negative N300 may reflect heightened emotional arousal to negative information as well as the processing of negative-relevant information. Similarly, our results that the more negative N300 effect was found for UPRIGHT TRIANGLE border might reveal that the border of UPRIGHT TRIANGLE had higher arousal strength and more negative valence to subjects, suggested that the UPRIGHT TRIANGLE was more suitable than CIRCLE to serve as the surrounding shape of the warning sign to deliver hazard information in order to affect the individuals' performance.

Thirdly, P300 represented many cognitive processes in different experimental processes or different tasks. One kind of the cognitive processes is the process of the cognitive evaluation of stimuli's meaning (Ito et al., 1998; Huang and Luo, 2006).

In the implicit-evaluation experiment, the process of evaluation on the non-target stimuli was implicit and automatic. The participant would inhibit the target-irrelevant information, in order to respond to the target stimuli more accurately. Especially, the inhibition of the negative valence information would be stronger. It has been vastly demonstrated that posterior P300 was an index of the inhibition process of target-irrelevant information (Campanella et al., 2002; Goldstein et al., 2002). The smaller amplitude of P300 represented stronger inhibition of the negative valence information (Yuan et al., 2007).

The result that the UPRIGHT TRIANGLE stimuli elicited smaller P300 than did the CIRCLE stimuli suggested that the UPRIGHT TRIANGLE evoked more uncomfortable feeling of the subjects than did the CIRCLE shape. The reason might be that the sharp angle of the TRIANGLE brings people an uneasy feeling and further an unsafe perception. In other words, the UPRIGHT TRIANGLE border delivered more hazard information to subjects than did the CIRCLE border.

Fourthly, LPP is another ERP component which may be elicited by emotional stimuli (Brown et al., 2012). In many cases, LPP is considered as a continuation of the evaluation process represented by P300. The amplitude of LPP is recognized as a reflection of the familiarity with the stimuli, such as own name (Symons and Johnson, 1997), or other self-relevant information (Miyakoshi et al., 2007). The more familiar with the stimulus image, the larger of the LPP is. Our findings were consistent with the previous studies (Symons and Johnson, 1997; Miyakoshi et al., 2007; Zhao et al., 2009). The CIRCLE shape is so common in daily life, such as wheels, cups, buckets, and dishes, that people are too familiar with it to cause alarm. While the UPRIGHT TRIANGLE shape is rarely seen in ordinary life. Once it appears, it is easy to attract attention, therefore, the warning sign with the UPRIGHT TRIANGLE border can easily convey the hazard information.

## Conclusion

The surrounding shape of UPRIGHT TRIANGLE had a greater attraction to attention (represented by P200), more negative valence and higher arousal level (represented by N300), and more uncomfortable/uneasy/unsafe information which was inhibited more strongly at the cognitive level (represented by P300 and LPP) when compared to the shape of CIRCLE, resulting in that the surrounding shape of UPRIGHT TRIANGLE was more suitable to use to deliver hazard information when designing a warning sign.

We believe that different borders have different warning effects and can be measured by electrophysiological indicators. At present, we are also conducting some extended research. The experiments we are doing have explored the interaction between multiple sign elements. Also for future research, we are going to study the comparison of various shapes, rather than simple triangles and circles. What's more, it's an interesting idea to assess the effect of different surrounding shapes on ERPs at a subliminal level, which could disentangle implicit and explicit effects.

## Ethics statement

This study was carried out in accordance with the recommendations from the ethics committee of Neuromanagement Lab, Zhejiang University. All subjects gave a written informed consent according to the Declaration of Helsinki. All participants had normal or corrected-to-normal vision. None of them reported any history of psychiatric or neurological disorders.

## Author contributions

QM and ZX conceived and designed the experiments. XB and GP performed the experiment. XB and GP analyzed the data. QM, XB, and GP wrote and refined the article.

### Conflict of interest statement

The authors declare that the research was conducted in the absence of any commercial or financial relationships that could be construed as a potential conflict of interest.

## Supplementary material

Supplementary Figure 1 Pictures are stimuli showed to participants, including non-target stimuli and target stimuli.
