# Supplementary figures and images for: The Hazard Perception for the Surrounding Shape of Warning Signs: Evidence From an Event-Related Potentials Study
Source: Front Neurosci. 2018 Nov 8;12:824. doi: 10.3389/fnins.2018.00824 (PMC6236016; doi:10.3389/fnins.2018.00824)

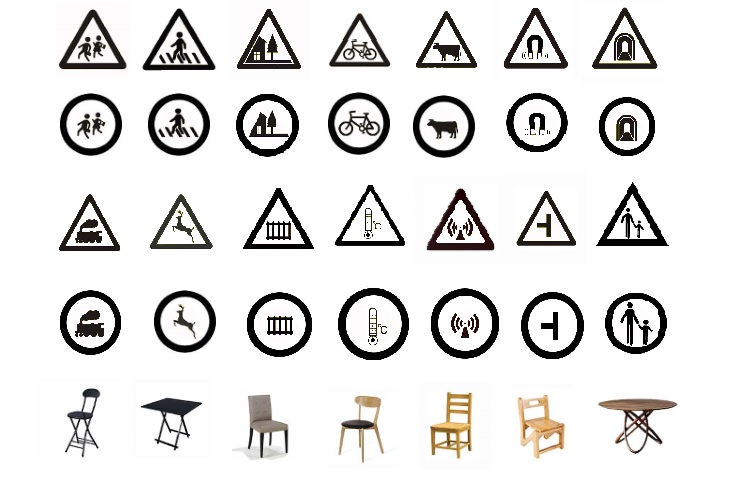

Supplement: Supplementary Figure 1 — Pictures are stimuli showed to participants, including non-target stimuli and target stimuli. [file Image_1.JPEG]
